# Digital sovereignty in public health

**DOI:** 10.2471/BLT.26.297133

**Published:** 2026-11-01

**Authors:** Marcelo D’Agostino, Ana Estela Haddad, Paula Xavier, Adriano Massuda, Alexandre Padilha, Jarbas Barbosa da Silva

**Affiliations:** a Pan American Health Organization, 525 23rd Street NW, Washington, DC 20037, United States of America (USA).; b Ministry of Health, Brasilia, Brazil.

The digital transformation of the health sector is a defining feature of modern public health. Electronic health records, telehealth platforms, disease surveillance systems, digital identity mechanisms and artificial intelligence (AI) increasingly shape how governments prevent disease, organize care, allocate resources and respond to crises. As digital transformation has become a central component of national policy agendas to strengthen health systems, digital sovereignty is increasingly important to governments and public health authorities.^1^

Digital sovereignty encompasses the capacity to exercise legitimate governance over digital technologies and services in accordance with applicable law and principles of transparency and accountability, while participating in an interconnected global digital system.^2^ This capacity includes establishing arrangements that ensure legal certainty, operational continuity, accountability, security, interoperability and public trust, regardless of the technology used.^2^ In public health, this governance helps protect citizens’ rights, sustain health services and support equitable innovation. 

Because health-related data are a strategic resource for public health policy-making and planning, data sovereignty also refers to the capacity of governments to establish the legal, ethical and governance conditions under which health data are collected, accessed, shared, reused and protected in accordance with national legislation and public health objectives. Health data derived from surveillance systems, mortality statistics, vaccination registries and epidemiological studies have long informed decision-making for better-quality health care. Health information systems have become critical health-care infrastructure and their protection now forms part of national public health security;^3^^,^^4^ therefore, they should be considered a public good. 

Protecting digital public health data is essential because without public trust, citizens and health workers may resist digital transformation. Globally, more than 130 jurisdictions now have privacy laws addressing personal data, including health information.^5^ These frameworks generally incorporate principles such as consent, transparency, purpose limitation, accountability and breach notification. 

Modern health systems integrate clinical data, as well as laboratory results, genomic information, data on pharmaceutical supply chains and cross-border human mobility, climate change, georeferenced data and, increasingly, patient-generated information from digital devices. These data sets enable earlier detection of outbreaks, better targeting of interventions, improved health service delivery and more efficient resource allocation.^6^ However, misuse or inadequate protection can expose individuals and institutions to harmful consequences, such as privacy violations, discriminatory profiling, algorithmic bias and systemic service disruptions caused by ransomware or data breaches.

To tackle these issues, governments have created national digital health policies to address regulatory gaps, safeguard interoperability and preserve public trust in digital public infrastructure. These policies vary across countries due to differences in legal traditions, economic conditions, political environments and health system frameworks, yet remain focused on practical and operational effectiveness.

Digital sovereignty should not be viewed as a single policy model but as a combination of complementary governance mechanisms. Brazil’s Unified Health System provides an example of how digital sovereignty can be translated into public policy. The Brazilian system depends on interoperable digital infrastructure to coordinate care across federal, state and municipal levels. The country’s National Health Data Network enables secure interoperability, continuity of care and nationwide exchange of health information while preserving governance, privacy and public accountability.^7^

However, effective digital health governance also requires institutional capacity, independent oversight, cybersecurity readiness and mechanisms that allow individuals to understand and influence how their data are used. Countries should also strengthen their capacity to manage the risks associated with overreliance on any single technology or provider while preserving innovation, interoperability and resilience.

Public health innovation increasingly relies on data-intensive technologies, including AI and predictive analytics. Sovereignty considerations influence how data sets are shared and used and, as AI becomes increasingly integrated into health systems, extend to AI governance. Sovereignty frameworks can support innovation models that respect governance and ethical standards. This point is especially relevant for countries with integrated health service delivery networks, as they generate large volumes of health data that are valuable for implementing AI models.^8^^,^^9^

The challenge for the coming decade is how digital sovereignty can be pursued in ways that reconcile national governance with international cooperation, autonomy with interoperability, resilience with innovation and public trust with the responsible use of emerging technologies. Digital transformation may deepen fragmentation or become a catalyst for stronger solidarity and cooperation. Achieving the latter requires governance innovation, technical cooperation and sustained dialogue grounded in mutual respect and shared responsibility.
